# Epidemiological and Ecological Characterization of the EHEC O104:H4 Outbreak in Hamburg, Germany, 2011

**DOI:** 10.1371/journal.pone.0164508

**Published:** 2016-10-10

**Authors:** Maike Tahden, Juliane Manitz, Klaus Baumgardt, Gerhard Fell, Thomas Kneib, Guido Hegasy

**Affiliations:** 1 Biometry and Data Management, Leibniz Institute for Prevention Research and Epidemiology—BIPS, Bremen, Germany; 2 Department of Psychology and Cluster of Excellence “Hearing4all”, Carl von Ossietzky University Oldenburg, Oldenburg, Germany; 3 Department for Statistics and Econometrics, University of Goettingen, Goettingen, Germany; 4 Division for Environmental Monitoring, Institut fuer Hygiene und Umwelt, Hamburg, Germany; 5 Centre for Infectious Diseases Epidemiology, Institut fuer Hygiene und Umwelt, Hamburg, Germany; University of Missouri Columbia, UNITED STATES

## Abstract

In 2011, a large outbreak of entero-hemorrhagic E. coli (EHEC) and hemolytic uremic syndrome (HUS) occurred in Germany. The City of Hamburg was the first focus of the epidemic and had the highest incidences among all 16 Federal States of Germany. In this article, we present epidemiological characteristics of the Hamburg notification data. Evaluating the epicurves retrospectively, we found that the first epidemiological signal of the outbreak, which was in form of a HUS case cluster, was received by local health authorities when already 99 EHEC and 48 HUS patients had experienced their first symptoms. However, only two EHEC and seven HUS patients had been notified. Middle-aged women had the highest risk for contracting the infection in Hamburg. Furthermore, we studied timeliness of case notification in the course of the outbreak. To analyze the spatial distribution of EHEC/HUS incidences in 100 districts of Hamburg, we mapped cases' residential addresses using geographic information software. We then conducted an ecological study in order to find a statistical model identifying associations between local socio-economic factors and EHEC/HUS incidences in the epidemic. We employed a Bayesian Poisson model with covariates characterizing the Hamburg districts as well as incorporating structured and unstructured spatial effects. The Deviance Information Criterion was used for stepwise variable selection. We applied different modeling approaches by using primary data, transformed data, and preselected subsets of transformed data in order to identify socio-economic factors characterizing districts where EHEC/HUS outbreak cases had their residence.

## Introduction

From May to July of 2011, a large outbreak of bloody diarrhea and hemolytic uremic syndrome (HUS) associated with entero-hemorrhagic E. coli (EHEC) occurred in Germany [1]. In this outbreak, 3,816 cases were notified to the Robert Koch-Institute (RKI), which represents the federal institution for disease prevention and control in Germany. The outbreak consisted of 2,971 cases of EHEC-gastroenteritis and 845 cases of HUS. During the outbreak, 54 fatalities were reported in Germany, 18 of which were EHEC cases and 36 were HUS cases. The causative agent in this epidemic was EHEC serotype O104:H4. As demonstrated by epidemiological studies, fenugreek sprouts served as a vehicle of infection [2]. A horticultural farm located in a distance of approximately 60 km from Hamburg was one source of EHEC contaminated sprouts [3]. According to RKI, this outbreak was the largest EHEC/HUS epidemic in Germany and considering the number of HUS cases, the largest outbreak worldwide to date [4].

The City of Hamburg was the first focus of the 2011 epidemic and amongst all 16 Federal States of Germany, it had the highest EHEC/HUS incidence [1]. Hamburg is divided into seven boroughs with one Local Health Department (LHD) per borough to carry out infectious disease surveillance and take outbreak control measures. According to the Infectious Diseases Law Reform Act (Infektionsschutzgesetz, IfSG), EHEC infection and enteropathic HUS are notifiable diseases in Germany [5]. Therefore, the laboratories identifying toxin-producing EHEC and the physicians diagnosing patients with enteropathic HUS have to report cases including their place of residence to the corresponding LHD. In Hamburg, case data are then transferred to the Center for Infectious Disease Epidemiology, a municipal division of the Institute for Hygiene and Environment (HU, Institut fuer Hygiene und Umwelt). After a review of case information, data are further transferred to RKI [6]. If Hamburg LHDs require support in their legal mandate, they are authorized to transfer personal data of cases to other public authorities including HU [7]. During the 2011 Hamburg EHEC outbreak, residential addresses of cases were transferred from LHDs to HU with the instruction to analyze case incidences at the level of city districts.

Based on these data, we analyzed the cases and mapped their addresses using geographic information software (GIS). We then questioned whether persons contracting EHEC/HUS and thus becoming cases were more likely to live in a district with a certain demographic or social background. As middle-aged women were particularly affected by the outbreak [8], we performed an ecological study to identify associations between socio-economic factors and EHEC/HUS incidences. Similar approaches have been applied for gastroenteric infections as campylobacteriosis, salmonellosis, rotavirus infection and giardiasis [9–11]. Furthermore infectious diseases as syphilis, gonorrhea, typhus and tuberculosis have been analyzed in this way [12–14]. In this context, we made use of the statistical report "Profiles of Hamburg Districts", which is published on a yearly basis by the Northern Bureau of Statistics (Statistikamt Nord) [15]. The report of this public-law institution includes 77 aggregated variables, which are classified into seven topics: population structure (20 variables), social structure (15 variables), city parliament election (7 variables), housing (14 variables), infrastructure (9 variables), transport sector (6 variables) and crime (6 variables). Using these variables as potential predictors and EHEC/HUS incidences as the outcome, we performed a stepwise Bayesian Poisson regression with structured and unstructured spatial effects. For fast and efficient inference, we applied the software R using integrated nested Laplace approximations (INLA) for posteriors [16]. Missing values were deduced from other survey years, or imputed as described in the methods section. We used the Deviance Information Criterion (DIC) for stepwise variable selection of the socio-economic covariates.

In this article, we present key epidemiological features of the 2011 EHEC outbreak in Hamburg and, furthermore, study the association between socio-economic parameters and case incidences on a district level by means of ecological regression analysis.

## Methods

### EHEC/HUS Outbreak Cases

Cases were evaluated using data from the electronic surveillance system by employing SurvNet2 software from RKI [6]. Case counts of the EHEC/HUS outbreak were based on the case definition as published by RKI on July 1, 2011 [4]. Thus, cases were included in the outbreak if the first onset of clinical symptoms were reported as early as May 1, 2011. For asymptomatic cases, the notification date was used instead. On July 26, 2011, the RKI announced that the outbreak had ended three weeks earlier as no cases had been reported with a symptom onset later than July 4, 2011. Case data and residential addresses were provided by the seven LHDs of Hamburg. Initially, 670 case notifications were available for our analysis. Nine cases were excluded because their data were incomplete. Furthermore, five duplicate cases were removed. Therefore, 656 cases were used in our study.

### Socio-Economic Variables of the Hamburg Districts

All data given in this article refer to the district map of Hamburg as it was in the year 2011 when the EHEC O104:H4 outbreak occurred. In this year, the City of Hamburg consisted of seven boroughs subdivided into 104 districts. The report "Profiles of Hamburg Districts, 2011" published by the Northern Bureau of Statistics contains 77 variables [15]. A complete list of these variables is given in supplementary S1 Table. In this report, four districts are merged with neighboring districts for data analysis as they are mainly industrial or agricultural areas with no or only very few inhabitants. This refers to Kleiner Grasbrook and Steinwerder, Waltershof and Finkenwerder, Neuland and Gut Moor, Moorburg and Altenwerder. Because the number of inhabitants per district is included in the disease incidence, 76 variables for 100 districts of Hamburg were available for our ecological analysis.

We used four variable pools in our study: 1) all 76 socio-economic variables, 2) partially transformed variables, 3) systematic preselection of partially transformed variables, and 4) knowledge-based preselection of partially transformed variables. The decision for variable transformation (none, logarithm, or square root) was based on consensus of independent decisions of two statisticians. This resulted in transformation of 48 variables as shown in S1 Table, column K. Furthermore, we applied two procedures of variable preselection. For the systematic variable reduction, the following exclusion criteria were applied: A) variables in absolute numbers without reference to number of inhabitants of a district, B) variables representing only a fraction of another variable already included in the analysis, C) variables from the categories "transport sector" and "crime". This resulted in 27 variables as shown in S1 Table, column L (including 7 transformed variables). For the knowledge-based variable preselection, we selected those variables from the report that we considered characteristic of the socio-economic background of the Hamburg districts. This decision was based on a consensus between an epidemiologist and a statistician. This protocol resulted in 11 variables which were used in our final analysis as shown in S1 Table, column M (including 6 transformed variables). To strengthen the validity of our analyses, we additionally wanted to include a method for dimension reduction as principal componenten analysis (PCA) or independent component analysis (ICA). As most density histograms of the 76 partially transformed socio-economic variables indicated approximate normal distributions, we chose PCA. After considering the scree plot and evaluating several possible solutions, seven principal components explaining 88% of the variance of the data were chosen. All 76 partially transformed socio-economic variables were assigned to one of the seven principal components with highest absolute loadings as shown in S1 Table, column N. A correlation analysis was performed between the partially transformed socio-economic variables and the principal components as shown in S1 Table, column O. We then compared correlation results from PCA with our systematic and knowledge-based preselection approaches 3) and 4), respectively.

### Ethics Statement

The national notification database hosted by RKI is a public use file [17]. Notification data on EHEC and HUS cases are collected in Germany according to IfSG [5]. The report "Profiles of Hamburg Districts, 2011" by the Northern Bureau of Statistics is publicly available [15]. Analysis of case data by the municipal Centre for Infectious Disease Epidemiology is covered by the Federal State Law on Public Health Services [7]. Therefore, approval by an ethics committee or written consent is not required.

### Geomapping

Analysis of the cases' district of residence and cartography was performed employing geographical information system software ESRI ArcGIS. Mapping was performed using the ArcGIS Address Locator Module and standard maps from the State Office Geoinformation and Geodetic Survey of Hamburg. Incidences were then calculated based on the number of inhabitants per district as published by the Northern Bureau of Statistics [15].

### Ecological Analysis Using Spatial Bayesian Poisson Regression

We performed a spatial disease mapping BYM model as described by Besag, York and Mollié, formulated in a hierarchical Bayesian framework [18]. This approach is in line with methods suggested by Schroedle et al. [19] and Wilking et al. [9]. We assumed that the number of EHEC/HUS cases ***y***_***i***_ is conditionally independent Poisson distributed with rate ***λ***_***i***_ per neighborhood ***i***:

$$y_{i}|\;\lambda_{i}\;∼Po\left(\lambda_{i}\right),\;i=1,\ldots ,100$$ (1)

$$log\left(\lambda_{i}\right)=\;log\left(m_{i}\right)+\;\mu \;+\;\psi_{i}\;+\;\nu_{i}\;+\;x_{i}^{\prime }\beta$$ (2)

Here, ***m***_***i***_ denotes the number of inhabitants divided by 10,000 that yields the offset **log(*m***_***i***_**)**. The intercept ***μ*** describes the overall level of the log risk rate. The structured and unstructured effects ***ψ***_***i***_ and ***ν***_***i***_, respectively, represent the area-specific spatial variation. Assuming that neighboring districts have similar incidence rates, the spatially structured component is modeled by ***ψ***_***i***_ as a Gaussian Markov random field (GMRF) with unknown variance $\sigma_{\psi }^{2}$. The spatially unstructured term ***ν***_***i***_ is assumed to follow a Gaussian distribution with zero mean and variance $\sigma_{v}^{2}$, which is included to allow for spatially uncorrelated heterogeneity. Furthermore, the covariate effects indicated by vector ***β*** denote the influence of the explanatory variables ***x***_***i***_ on the outcome_._ The regression coefficients **(*μ*,*β*')'** are assumed to follow a zero-mean Gaussian prior with variance $\sigma_{\beta }^{2}$. Finally, for the unknown hyperparameters ***σ***_***β***_, ***σ***_***ψ***_, ***σ***_***ν***_ we assume ***Ga(a*,*b)*** hyperprior distributions that are conjugate to the Gaussian prior for the corresponding effects. For model fitting, we used INLA proposed as an approximate, fast and efficient Bayesian inference method for latent Gaussian models by Rue et al. [16]. In the software R, the R-INLA packages implements the procedures [20]. We used the software version R-3.2.2 and scaled the spatial effect to ensure fixed hyperpriors for the precision of all GMRFs in the model [21].

### Missing Values and Imputation

Among the socio-economic variables published in the "Profiles of Hamburg Districts, 2011", 13 variables contained missing values (for details see S1 Table**,** column J). In the category social structure, 9 variables related to unemployment and social welfare contained 33 missing values (variables from No. 26 to No. 34). According to the Northern Bureau of Statistics, data variables with counts below three were censored by means of data privacy protection and prevention of identification of individuals. Consequently, we complemented these missing values with 1.5 (or 1 and 2 where applicable). Furthermore, two values for variable No. 35 (average income per tax payer) were missing. Since the missing district values were included in the 2007 survey, we were able to estimate the missing values from 2004 based on the average income development in Hamburg. In the category housing, 82 values were missing for three variables (No. 54, No. 55, No. 56). These variables represent average square meter prices for different types of real estates. Thus, values were missing when too few transactions had taken place in 2010. As values from 2009 to 2014 were available for these three variables, we interpolated the prices considering the average price development in the real estate type and/or district. Finally, this procedure yields nine missing values in the data set. Assuming that these values are missing completely at random (MCAR) or missing at random (MAR), the application of imputation methods is possible. In this context, we performed regression imputation as proposed by Yates [22]. The approach is based on regression methods using the variable containing the missing data as the response variable and variables with complete data as potential predictor variables. For variable selection, we applied the stepwise regression algorithm as described below. For target variables No. 54, No. 55 and No. 56, we assumed a ***Ga(a*,*b)*** distribution ignoring an offset **log(*m***_***i***_**)**. Furthermore, missing values were replaced by predictions. By performing this single imputation method we could also use a Bayesian approach for inference, exploiting our data volume and taking advantage of the multicollinearity between the variables.

### Stepwise Variable Selection

Bayesian hierarchical models are commonly compared by DIC, that measures the trade-off between model fit and model complexity [23]. It represents a commonly employed criterion for comparing Bayesian hierarchical models. Using INLA, the DIC can be easily obtained [16]. We implemented a stepwise regression algorithm using DIC for variable selection, called stepDIC. Our algorithm proceeds as follows: it starts with a null model containing only the offset, intercept and spatial effects

$$log\left(\lambda_{i}\right)=\;log\left(m_{i}\right)+\;\mu \;+\;\psi_{i}\;+\;\nu_{i}.$$ (3)

We define a pool of potential explanatory variables. Subsequently, the algorithm alternates forward and backward variable selection steps until it selects the best model according to DIC. We tested the robustness of our implemented stepDIC algorithm by comparing its results with those from standard stepAIC procedure for likelihood-based generalized linear models, and came to similar results.

## Results

### Epidemiological Key Figures

**Fig 1:**
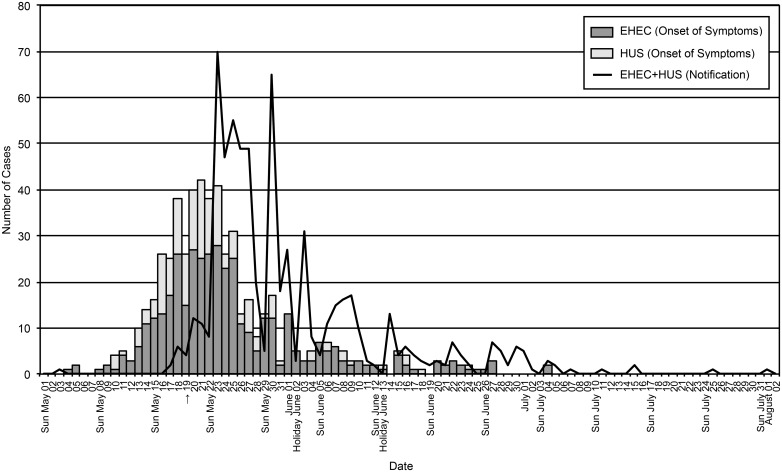
Epicurve of EHEC O104:H4 Outbreak in Hamburg, Germany. Shown are EHEC and HUS cases according to date of symptom onset (boxes, n = 656) and notification date (line, n = 554). The last outbreak-associated case fell ill on July 4, 2011. The arrow indicates the day when RKI was informed of a cluster of three pediatric HUS cases admitted to the Hamburg University Hospital.

In the 2011 EHEC/HUS outbreak, the City of Hamburg had been the first focus of the epidemic, and highest incidences throughout the outbreak among all 16 Federal States of Germany were reported here. Thus, we aim to present key figures of the notification data from Hamburg as a hotspot of the EHEC epidemic in Germany. Based on the SurvNet notification data, we counted 656 cases for the outbreak in Hamburg in accordance with the case definition. The data consist of 174 HUS cases and 482 EHEC cases. The overall incidence for Hamburg was 37.6 per 100,000 inhabitants, comprising an incidence of 27.6 for EHEC and 10.0 for HUS. For all 656 cases, a date of notification was present in the surveillance data. For 554 cases, a date of symptom onset was available. For 102 cases, the information on symptom onset was not available. In 58 cases, symptoms consistent with the outbreak case definition were present, but the onset date could not be defined. Thirty-nine cases were asymptomatic at the time of notification. For the remaining 5 cases, information on presence or absence of symptoms was missing entirely. Thus, an epicurve was plotted for all 656 EHEC/HUS cases based on the notification date and a second epicurve for 554 cases based on the date of symptom onset. Fig 1 shows these epidemic curves with the arrow indicating the date when the initial outbreak alarm was triggered. On this day the LHD of Hamburg-North was informed about three pediatric HUS cases presenting on the same day to the children's department of the University Hospital Hamburg-Eppendorf. This information was reported to RKI, and as a consequence, an outbreak investigation team was sent [2].

**Fig 2:**
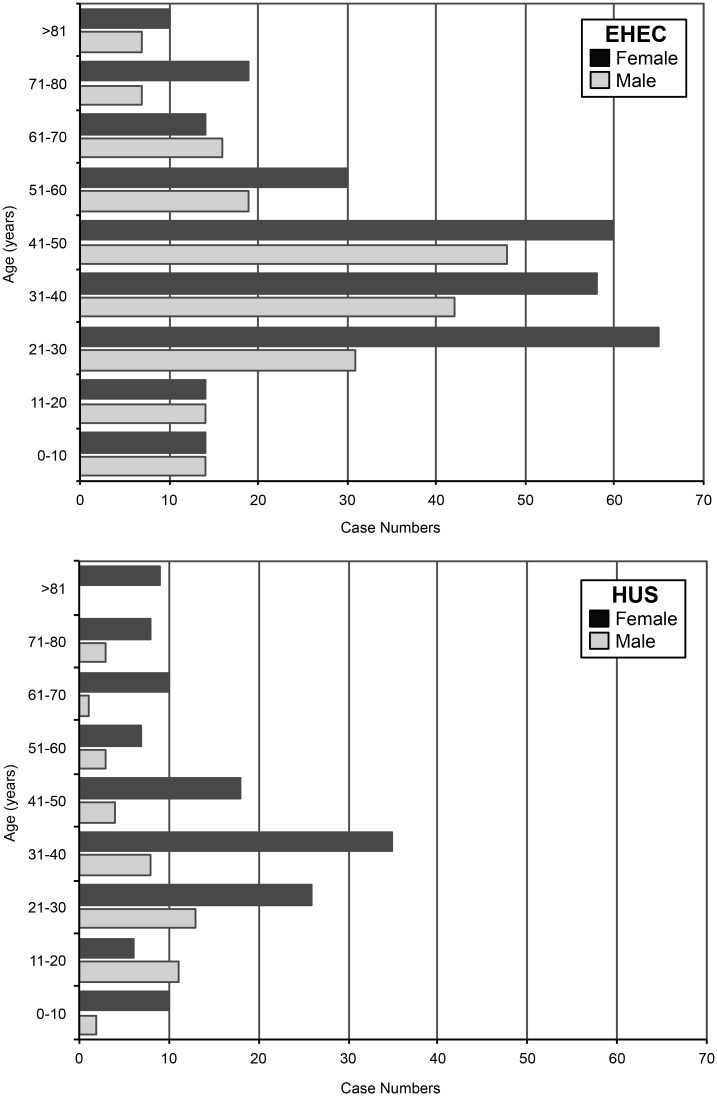
Age and Sex Distribution of EHEC and HUS Cases during EHEC O104:H4 Outbreak in Hamburg, 2011. Nine age groups were defined for EHEC cases (upper panel, n = 482) and HUS cases (lower panel, n = 174).

It has been an early epidemiological finding in the course of this outbreak that demographic characteristics were different from EHEC/HUS notifications in earlier years [8,24]. This can be confirmed by the Hamburg dataset. In the years 2005 to 2010, 21 cases of HUS were notified in Hamburg. All patients were children and the sex distribution was balanced [17]. During the outbreak in 2011, the average age of HUS cases was 38.5 years (median 34.0) and 40.4 years (median 38.5) for EHEC cases. As shown in Fig 2, women were predominantly affected by the outbreak. 62.9% of EHEC cases and 74.1% of HUS cases were female. Women in the age group of 31–40 years had the highest risk to become infected in the outbreak.

The notification data of the 2011 EHEC/HUS outbreak have been considered suitable to evaluate the timeliness of the surveillance system in Germany [25]. We analyzed the Hamburg data in order to calculate the time interval between symptom onset and the notification date on the basis of the corresponding calendar week. Results are shown in Table 1. In the first two weeks of the outbreak, the median latency between onset of symptoms and receipt of a notification by an LHD in Hamburg was four days. During the peak of the outbreak in the 21^st^ calendar week, the notification timeliness remained stable. As shown, latency between symptom onset and disease notification increased in the 22^nd^ week to five days, and in weeks 23 and 24 to six days. While numbers of cases notified steeply decreased from the 22^nd^ week onwards, notification latency peaked in week 25 to seven days and fell back to five days in the 26^th^ calendar week.

**Table 1:** Interval between Symptom Onset and Case Notification from Calendar Week 20 to 26.

Calendar Week	20	21	22	23	24	25	26
Date	16. - 22. May	23. - 29. May	30. May—5. June	6. - 12. June	13. - 19. June	20. - 26. June	27. June—3. July
Cases	43	268	127	57	21	11	19
Q1	2,5	3	3	3	4	4	4
Med	4	4	5	6	6	7	5
Q3	6	7	9	14	8	14	12

Cases = Number of notified cases per calendar week (n = 546), Q1 = 1st Quartile, Med = Median, Q3 = 3rd Quartile.

Hospital-associated transmissions of infectious diseases may play an important role in an outbreak situation. During the 2011 EHEC epidemic it has been reported that a nosocomial transmission was observed in the Federal State of Hesse [26]. Furthermore, in the post-outbreak phase a transmission in a hospital following a diagnostic procedure has been reported [27]. According to our surveillance data, no nosocomial infection occurred in Hamburg in the 2011 EHEC outbreak. However, such an incident could not be formally excluded, because the legal obligation to transfer data on nosocomial infections from LHD via HU to RKI became effective one month after the outbreak had ceased (August 4, 2011).

**Fig 3:**
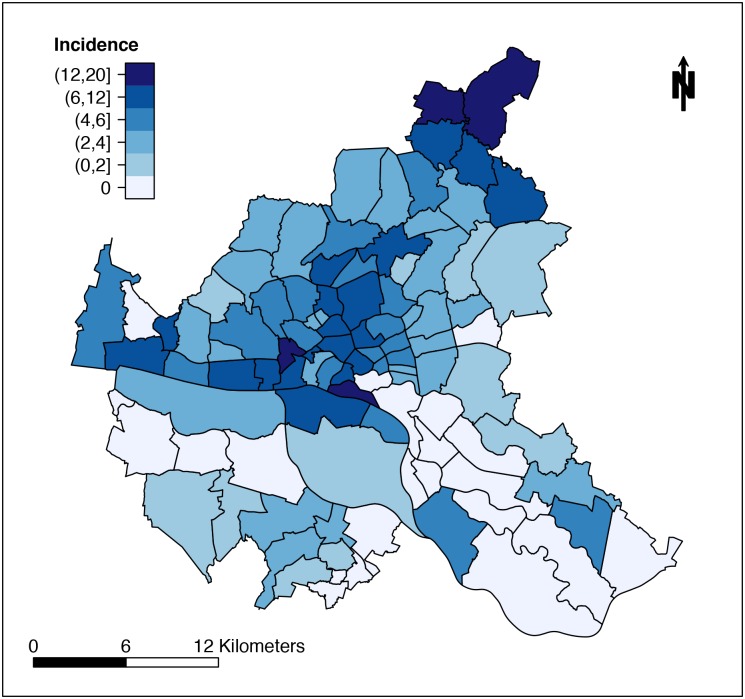
Spatial Distribution of EHEC/HUS Incidences of Hamburg during EHEC O104:H4 Outbreak 2011. Street addresses of EHEC/HUS cases were mapped and incidences (cases per 10,000) calculated for 100 districts of Hamburg. Incidences were categorized in six groups as shown.

**Fig 4:**
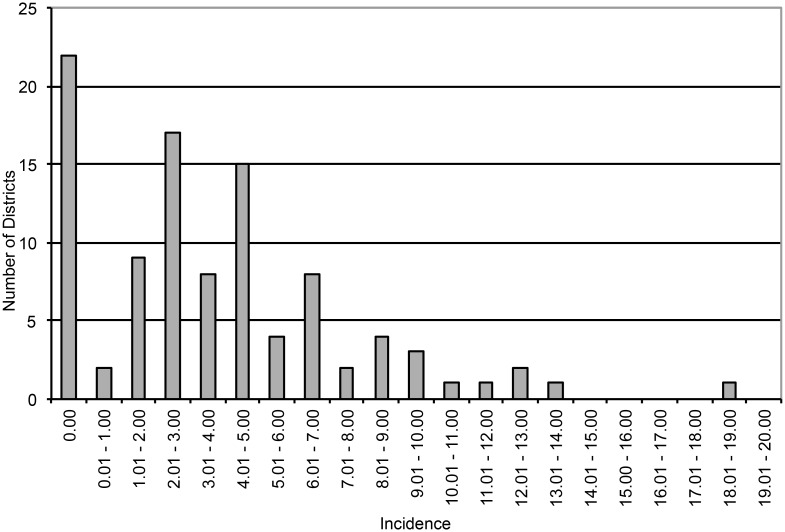
EHEC/HUS Incidence Distribution in 100 Districts during EHEC O104:H4 Outbreak in Hamburg, 2011. Incidences (cases per 10,000) were categorized in 21 groups as indicated.

In order to analyze the incidence of EHEC and HUS cases on a district level, we mapped residential addresses of cases using the ESRI ArcGIS address locator module. In this process, 99% of the cases' addresses had a perfect match. However, for 1% an equivalent candidate address was suggested by the software based on neighboring house numbers. For district-specific incidences cases per 10,000 inhabitants were calculated based on number of residents as published in the "Profiles of Hamburg Districts, 2011". Fig 3 shows the case incidences of the EHEC O104:H4 outbreak on the map of Hamburg. Distribution of incidences among 100 districts of Hamburg is shown as a barplot in Fig 4. The incidence ranges from zero (22 districts) to 18.23 (one district).

### Ecological Analysis

In order to understand the socio-economic background of the districts where EHEC/HUS outbreak cases had their residences, we performed an ecological study. We analyzed variables as covariates for the EHEC/HUS incidences on a district level. In particular, we performed an ecological regression using a spatial Bayesian Poisson model. Variables with explanatory value were selected by a data-driven stepwise DIC algorithm. Based on the data published in the report "Profiles of Hamburg Districts, 2011" we applied four variable pools: 1) all available variables, 2) all variables transformed by log or square root where applicable, 3) 27 systematically preselected and transformed variables, and 4) 11 knowledge-based preselected and transformed variables. Results of the first and the last model are shown in Table 2. Results of both intermediate models are shown in S2 Table. Tables show the covariates in the order they were selected by the stepDIC algorithm, the corresponding DIC value and excess risk ratio (ERR), as well as the 95% Bayesian credibility interval (CI). The first DIC value of 454.80 corresponds to the regression model containing only the offset, intercept and spatial effects.

**Table 2:** Explanatory Variables in Poisson Models for EHEC/HUS Outbreak Incidences.

**A**				
No	Variable	DIC	ERR	95%CI
-	-	454.80	-	-
37	Turnout of voters, city parliament election 2011	439.62	7.25	(5.72, 8.85)
14	Proportion of single-person households to all households in percent	420.71	2.59	(1.71, 3.51)
7	Proportion of foreigners to all inhabitants in percent	418.71	3.24	(1.02, 5.42)
74	Number of violent offences	418.30	-0.04	(-0.09, 0.01)
**B**				
No	Variable	DIC	ERR	95%CI
-	-	454.80	-	-
37	Turnout of voters, city parliament election 2011	439.62	7.05	(4.62, 9.55)
14	Proportion of single-person households to all households in percent	420.71	2.76	(1.58, 3.97)
55	Average price for real estate in EUR per square meter	422.22	-19.68	(-47.39, 22.90)
7	Proportion of foreigners to all inhabitants in percent	420.85	49.86	(6.18, 111.50)
49	Average living space per inhabitant in square meter	420.08	1.77	(1.23, 4.22)

DIC = Deviance Information Criterion, ERR = excess risk ratio, i.e., (RR-1) 100%, 95%CI = Bayesian 95% credibility interval. **(A)** initial model using 76 primary variables without transformation or selection. **(B)** final model using a subset of 11 variables after transformation where appropriate.

The first approach including all 76 untransformed variables (Table 2A) resulted in a model containing variables No. 37 "turnout of voters in percent", No. 14 "proportion of single-person households to all households in percent", and No. 7 "proportion of foreigners to all inhabitants in percent". In this context, an ERR = 7.25 can be read as follows: for each percent increase in turnout of voters, the EHEC/HUS incidence is expected to increase by 7.25%. Additionally, variable No. 74 "number of violent offenses" is included in this model, but the 95%CI contained zero.

In the next two approaches, variables were first transformed where appropriate and then the number of variables was systematically reduced. When using 76 partially transformed variables the resulting model contained 9 variables starting with 5 variables where the 95%CI contained zero (S2 Table). Variables where the 95%CI did not contain zero were No. 42 "percentage of valid votes for FDP (Free Democratic Party), city parliament election 2011", No. 26 "number of registered unemployed between 15 and 25 years of age", No. 43 "number of residential buildings" and again No. 37 "turnout of voters". When using a systematic preselection of 27 partially transformed variables, the resulting model started with two variables where the 95%CI contained zero (S2 Table). As a positively associated factor variable No. 42 "percentage of valid votes for FDP (Free Democratic Party), city parliament election 2011" was included, and No. 38 "percentage of valid votes for CDU (Christian Democratic Union), city parliament election 2011" as a negatively associated factor. No. 37 "turnout of voters" was included in this model, but the 95%CI contained zero.

**Fig 5:**
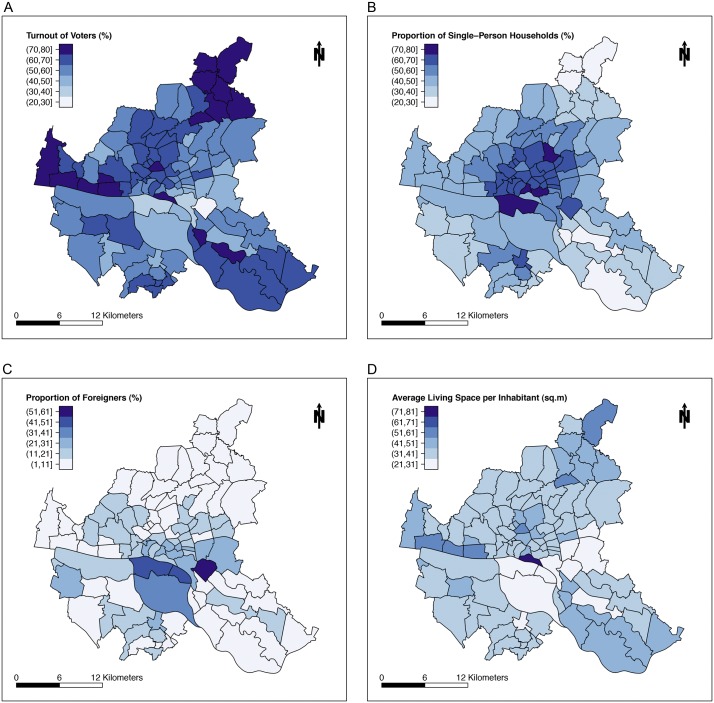
Spatial Distribution of Four Socio-economic Variables in 100 Districts of Hamburg. Fig 5A-D show distribution maps for explanatory variables from models in Table 2. **(A)** variable No. 37 “turnout of voters, city parliament election 2011”, **(B)** variable No. 14 “proportion of single-person households to all households in percent”, **(C)** variable No. 7 “proportion of foreigners to all inhabitants in percent”, **(D)** variable No. 49 "average living space per inhabitant in square meter".

For our fourth model, we used a reduced variable pool such that it contained only 11 partially transformed variables**.** As shown in Table 2B, with this subset we found a model resembling the results of our initial approach that contained all 76 untransformed variables. In this model the DIC values did not decrease continuously, because the algorithm first selected two variables that were removed in subsequent iteration steps. Again variables No. 37 "turnout of voters in percent", No. 14 "proportion of single-person households to all households in percent", and No. 7 "proportion of foreigners to all inhabitants in percent" were included in the model. Variable No. 55 "average price for real estate in EUR per square meter" was chosen by the algorithm, but the 95%CI contained zero. As an additional positively associated factor, variable No. 49 "average living space per inhabitant in square meter" was included in this model. Fig 5A–5D show maps of Hamburg for the socio-economic variables included in our models specified in Table 2A and 2B.

**Fig 6:**
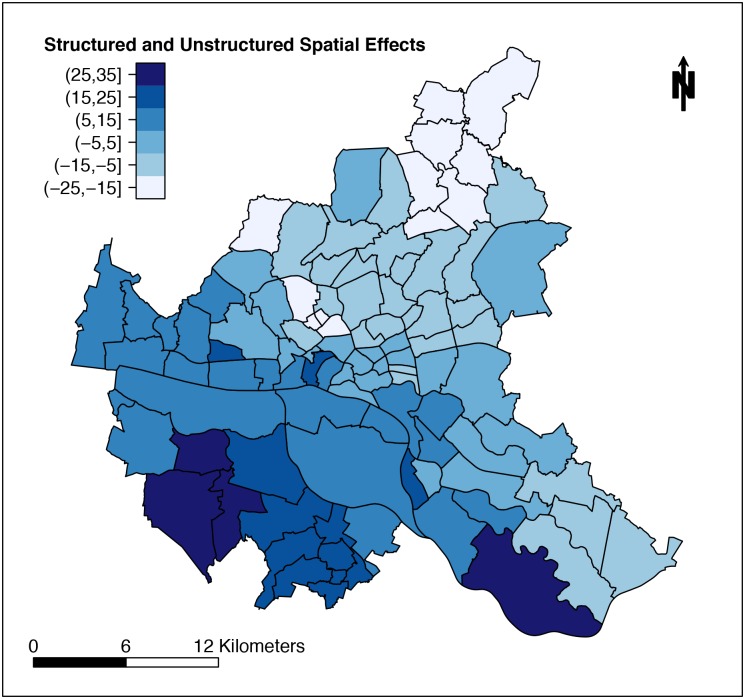
Structured and Unstructured Spatial Effects of Regression Model in 100 Districts of Hamburg. Shown is the excess risk ratio due to combined structured and unstructured spatial effects from model in Table 2A, i.e., exp((*ψ*_*i*_ + *v*_*i*_) −1) ∙ 100.

**Fig 7:**
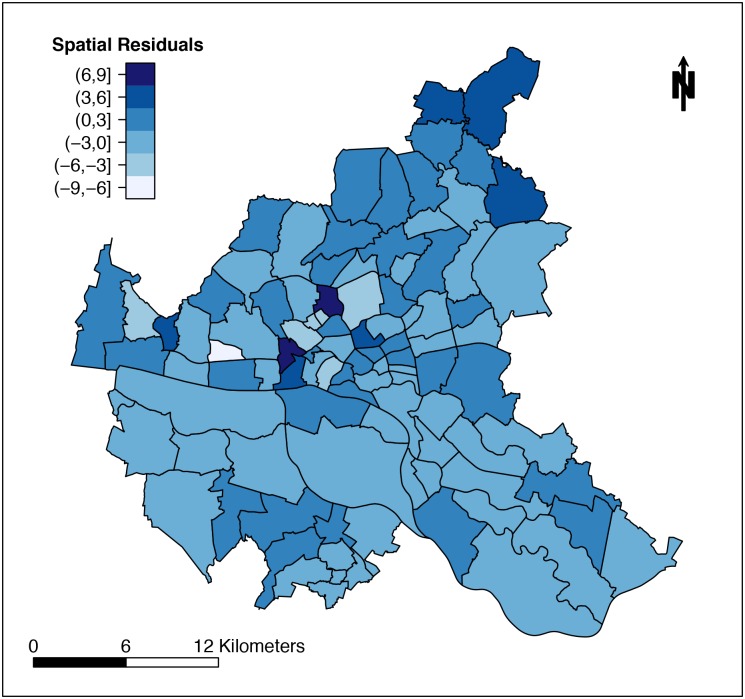
Spatial Residuals of Regression Model in 100 Districts of Hamburg. Shown is the difference between EHEC/HUS cases and those estimated by the model in Table 2A, i.e., $\left(y_{i}+{\hat{y}}_{i}\right)$ per neighborhood *i*.

When comparing results of PCA to our variable preselections, we found that the first component was represented with only one variable in the data pool of 27 variables (third approach) and with no variable in the data pool of 11 variables (fourth approach). Thus, we decided to complement our modelling analyses with two variables showing highest correlation from the first principal component. When including these variables (No. 2, No. 36), the resulting models remained unchanged. To test the model assumption that neighboring districts have similar incidences, we additionally fitted all four approaches without the spatially structured component ***ψ***_***i***_ modelled as a GMRF (cf. (Eq 2) in methods section). All four DIC values were higher indicating inferior model performances (first model without ***ψ***_***i***_ DIC = 430.10, second model without ***ψ***_***i***_ DIC = 424.42, third model without ***ψ***_***i***_ DIC = 421.90, fourth model without ***ψ***_***i***_ DIC = 425.53). The spatial effect combining structured and unstructured spatial ERR of our first regression model exhibits a smooth pattern from northeast to southwest as shown in Fig 6. We also plotted the residuals from this regression model in 100 neighborhoods in Hamburg as shown in Fig 7. Neither specific clustering nor particular observable structuring can be detected, which indicates that the model assumptions are fulfilled.

## Discussion

In this article, we give an epidemiological description of the Hamburg EHEC/HUS outbreak in 2011. As shown in the epicurve, number of notifications started to increase in Hamburg from May 17 to 22, 2011 and steeply rose on May 23, 2011. On May 19, 2011 the RKI was informed about three pediatric HUS cases that were admitted to the University Hospital Hamburg-Eppendorf. This was interpreted as an epidemiological signal and the RKI sent an outbreak investigation team [2]. It has been a matter of debate if the epidemiological signal would have been detectable solely from the surveillance data [25]. According to our data, until the evening of May 18, 2011 seven HUS cases and two EHEC cases were notified in Hamburg. However, since they were reported to five different LHDs, it was unlikely that this could have led to an epidemiological alert on the level of the boroughs of Hamburg. According to the version of the IfSG effective in 2011, data transmission between LHD and HU had to take place at the latest on the third day of the week following the notification date. Thus, an epidemiological signal might have been detectable on the municipal level, but information about the notified cases had not reached HU at that point in time. Only the direct observation by a pediatrician of three HUS patients admitted to the children's department of the University Hospital Eppendorf could result in an alert on May 19, 2011. This is in line with Altmann et al., who found that the identification of an epidemiological signal would have been considerably later, if the LHD in charge had not transmitted the information on the HUS case cluster to the RKI [25]. When the epidemic became evident, already 147 patients experienced their first symptoms, but were diagnosed and notified later. Of the 147 cases, 99 were diagnosed with EHEC (20.5% of the total EHEC outbreak cases) and 48 with HUS (27.6% of the total HUS outbreak cases). But it was not until May 23, 2011, when based on the notification data the extent of the epidemic became evident in Hamburg. The frequency of notification data transmission from LHDs to the Federal State level and then subsequently to RKI was reduced to 24 hours because of a new law from March 2013 [28].

Altmann et al. have analyzed the timeliness of surveillance in Germany in this outbreak [25]. For the period between symptom onset and report to an LHD, they found a median of five days. This comprises the time between first symptoms, visit to a physician, laboratory and/or clinical diagnosis, and notification to the LHD in charge. For our data from Hamburg, we found a median latency of four days for the 20^th^ and 21^st^ calendar week and a latency of five days for the 22^nd^ calendar week. During this period 72.3% of all cases of the outbreak were notified. The notification latency increased in week 23 and 24 to six days and to seven days in week 25 when only 10.4%, 3.8% and 2.0% of all cases were reported, respectively. This indicates that the timeliness of EHEC/HUS surveillance in Hamburg was not delayed by the enormous amounts of notifications at the peak of the outbreak. We can only speculate what the other factors were that led to the delay of notification in the later course of the outbreak.

It has been an early epidemiological finding that demographic characteristics in this outbreak differed from earlier EHEC/HUS notification data [8,24]. This holds true for the Hamburg data set as well. The average age of EHEC cases here was 40.4 years (median 38.5) and 38.5 years for HUS cases (median 34.0). Furthermore, middle-aged women had the highest risk of contracting the infection during the outbreak in Hamburg. Frank et al. speculated that this was a result of sprouts as food vehicle [1]. They suggest that middle-aged women were more likely to contract the infection because they are more likely to consume sprouts. This was attributed to women having a generally higher health consciousness, with sprouts being considered as a particularly healthy food. In order to add to this picture, we conducted an ecological study with socio-economic parameters as the explanatory variables, and incidences as the target variable. Similar approaches have been applied for example for gastroenteric infectious diseases caused by rotavirus, campylobacter, salmonella, and giardia [9–11]. However, these studies analyzed endemic situations, while we employed this approach for a disease outbreak. Wilking et al. studied rotavirus gastroenteritis in Berlin, Germany based on notification data similarly to ours surveyed according to IfSG [9]. They identified variables such as "percentage of unemployed inhabitants" and "percentage of children attending day care attendance" in a neighborhood as associated risk factors for infants to contract the infection. Ecological analyses are used when data are not accessible on an individual level (e.g., for data privacy regulations), and may give indications for risk factors on the basis of aggregated disease counts. A general limitation of these analyses is the potential for ecological fallacy [29]. Furthermore, many of the socio-economic variables in our study were given as percentages, which bears the risk of spurious correlations. However, we consider our models as suggestions for hypothesis generation keeping in mind that the cases themselves are not described, but the residential environment associated with case occurrence.

In our basic approach, all 76 variables without transformation or selection were used for modeling. Positively associated factors were "turnout of voters", "proportion of single-person households to all households", and "proportion of foreigners to all inhabitants". This model appears to give an image of a neighborhood, where high voter participation might indicate presence of a politically interested, educated civic stratum. An increased proportion of foreigners might point to districts, where foreign food is consumed more often. This seems reasonable since fenugreek is an exotic food in Germany, but is a regular ingredient in other cultures especially found in Arabic, African and Asian cooking. Increase of single-person households in a neighborhood that was previously populated by foreigners may occur in association with gentrification. Since both variables were included in the model, we speculate that sprout consumers in part had their residence in areas affected by such processes. The spatial effect of this model exhibits a pronounced increase of excess risk rate in the northeast to southwest direction. These differences could be explained by missing predictor variables which exhibit their own spatial structure.

When we used 76 partially transformed variables, a model resulted that exhibited the lowest DIC value of all four approaches. A low DIC value can occur when a large number of less influential variables is included, thereby maximizing the explanatory power of the model. In this model, nine variables were incorporated, but only four of them had effects where the 95%CI did not contain zero. We find "percentage of valid votes for FDP" (Germany's liberal party), "unemployed between 15 and 25 years of age" and again "turnout of voters" as positively associated factors. It is more difficult to deduce an improvement for the variable "number of residential buildings" as a negatively associated factor.

In the third approach, we reduced the variable pool to 27 variables by systematic preselection of the variables. In the resulting model the 95%CI contained zero for three out of five variables. As a positively associated factor "percentage of valid votes for FDP" was included in this model, while "percentage of valid votes for CDU" (Germany's conservative party) was negatively associated. These two variables may fit to the picture of districts that are populated by a liberal, civic stratum as interpreted from the first model.

In our fourth approach, we used a reduced variable pool by knowledge-based preselection containing 11 partially transformed variables. From the report, these 11 variables were considered the most suitable to characterize the socio-economic background of districts in Hamburg. Similar to our first model, positively associated factors where 95%CI did not contain zero were again "turnout of voters", "proportion of single-person households", and "proportion of foreigners". In this model, the picture was further extended by the factor "average living space per inhabitant" that was positively associated. We speculate that an increased average living space per inhabitant points to districts of Hamburg where inhabitants are rather well-off.

Our ecological study is intended to find associations between socio-economic characteristics of neighborhoods in Hamburg and EHEC/HUS 2011 outbreak incidences. With this approach we wanted to add to the picture that emerges from the fact that middle-aged women were overrepresented in the outbreak and fenugreek sprouts served as a vehicle of infection. It is tempting to speculate what hypothesis would have been drawn if our study had been performed while the outbreak was taking place. When the nature of the outbreak source is entirely unclear, an approach including all 76 primary variables might be chosen. When assuming a food vehicle that might be consumed only by a fraction of the population whose residence background can be characterized by available socio-economic variables, our approach using knowledge-based preselected variables might have been employed. In this context it is remarkable, that both approaches resulted in similar models. It is conceivable that the results of these models would have been interpreted in a way that the infection vehicle might rather be an unconventional food as we consider fenugreek sprouts to be, than conventional food, e.g., commonly consumed vegetables. However these considerations remain largely speculative and can only be seen as suggestions for hypothesis generation.

When interpreting the socio-economic covariates it is important to keep in mind that our statistical models do not control for possible bias and confounding. Furthermore, they do not reflect the contagious nature of the disease. Over-interpretation has to be avoided, and all hypotheses drawn on aggregated data would have to be analyzed in an individual-based study involving direct contact and questioning of cases.

We compared the results from PCA with our systematic and knowledge-based preselection approaches, and subsequently complemented our variable pools with two major variables from the first PCA component. However, the resulting models remained unchanged, supporting our preselection approaches. We decided to focus our work on these approaches rather than PCA as we wanted to take into account possible directional relationships between the outcome and the potential predictor variables. The statistical model framework can be extended in various directions. First, incorporating non-linear effects may allow for more flexible modeling of the covariates. However, this also reduces the power of the model. Second, district-specific reporting behavior could be captured by an additional spatial variable. Last, the model could be extended to a spatio-temporal model that considers the temporal trend of the infection reports. However, we wanted to keep the complexity of the model within reasonable bounds to avoid overfitting. In summary, taking into account the overrepresentation of middle-aged women and fenugreek sprouts as a vehicle of infection, our ecological study adds to the picture of the socio-economic background of the districts where cases of the 2011 EHEC/HUS outbreak had their residences.

## Supporting Information

S1 Table List of all Socio-economic Parameters of the "Profiles of Hamburg districts, 2011". Parameters were translated to English from original publication in German of the Northern Bureau of Statistics (Statistikamt Nord). Column J, "NA" = Missing Values. Column K, "Transf." = Transformation by taking the logarithm (log) or square root (sqrt) as indicated. Column L, "Select. 27", Column M, "Select. 11" = Selected variables as described in the methods section. Column N, “Assign. PC” = Assignment to the seven resulting principal components of PCA as described in the methods section, Column O, “Corr” = Pearson correlation between principal components and the partially transformed socio-economic variables (cf. column K).
(XLSX)

S2 Table Explanatory Variables in Two intermediate Poisson Models for EHEC/HUS Outbreak Incidences. DIC = Deviance Information Criterion, ERR = excess risk ratio, 95%CI = Bayesian 95% credibility interval.
(XLSX)
